# Water deficit modifies C:N:P stoichiometry affecting sugarcane and energy cane yield and its relationships with silicon supply

**DOI:** 10.1038/s41598-021-00441-0

**Published:** 2021-10-22

**Authors:** Antonio Santana Batista de Oliveira Filho, Renato de Mello Prado, Gelza Carliane Marques Teixeira, Marisa de Cássia Piccolo, Antonio Márcio Souza Rocha

**Affiliations:** 1 Department of Agricultural Production Sciences, São Paulo State University (UNESP), Via de Acesso Prof. Paulo Donato Castellane, s/n, Jaboticabal, São Paulo 14884900 Brazil; 2 Center of Nuclear Energy in Agriculture (CENA), University of São Paulo (USP), Piracicaba, São Paulo Brazil; 3 Department of Technology, São Paulo State University (UNESP), Jaboticabal, São Paulo Brazil

**Keywords:** Physiology, Plant sciences, Climate sciences

## Abstract

Climate change has increased the occurrence of water deficit in regions where sugarcane and energy cane are cultivated, jeopardizing dry matter production of stems. It was hypothesized that the reasons behind this fact relate to C:N:P stoichiometric modifications in these species that impair the conversion rates of accumulated nutrients in the stems, which could be attenuated by supplying silicon (Si) to the crops. Thus, the aims of this study were to evaluate the effects of water deficit in sugarcane and energy cane ratoons in the presence and absence of Si, in the C:N:P stoichiometry of stems, in the use efficiency of these nutrients and in the accumulation of dry matter in stems. Two experiments were carried out, using sugarcane (*Saccharum officinarum*) and energy cane (*S. spontaneum*), cultivated in pots filled with a Typic Quartzipisamment. The treatments for both experiments were arranged in a factorial scheme 2 × 2, without (70% of the soil’s water retention capacity) and with (30% of the capacity) water deficit, without and with the application of Si via fertirrigation, associated with foliar pulverization, both at a concentration of 2.5 mmol L^−1^, arranged in randomized blocks. The reduction in dry matter production of stems in both species caused by water deficit was due to modifications of the C, N and P stoichiometric homeostasis, but the benefit of Si in these plants when increasing dry matter production was not a reflection of the change in homeostasis, thus it may be involved in other mechanisms that remain unknown and should be further studied.

## Introduction

The sugar-energy industry sector is responsible for the widespread cultivation of sugarcane (*Saccharum officinarum* L.), which is a plant species with high concentrations of sucrose used for the production of sugar and ethanol, a biofuel that is widely used in Brazil. However, in view of the sector’s need to expand the cogeneration of electrical energy, the insertion of new species has been verified, such as cane energy (*S. spontaneum* L.), which typically presents high concentrations of fiber, a low sucrose content and high biomass yield^1^.

Sugarcane and energy cane are commonly exposed to water deficit conditions throughout its development^2^, seen that climate change has been causing extreme droughts in different regions where these species are cultivated, in higher frequencies over the last decades. Water deficit in plants is more alarming in regions with sandy soils of low water retention capacity^3^, which can lead to significant damages in the development and dry matter production of both species^4^.

Several studies on the damages caused by water deficit in sugarcane have been conducted, while reports on energy cane are scarce. In addition, these studies are restricted to the effects of water deficit on the gas exchange assessed in leaves^5,6^, although the greatest share of the biomass produced by sugarcane comes from the stems, which can be severely impaired and end up compromising the crop’s productivity. There is evidence that water deficit can reduce the absorption of nutrients accumulated in the aerial part, especially N and P, in other species such as *Hordeum vulgare, Zea mays, Andropogon gerardii*^7^ and *Punica granatum* L^8^.

Recent and innovative studies on water deficit have indicated some modifications in the C:N:P stoichiometry of distinct plant species, such as *Zygophyllum xanthoxylum*^9^, *Panicum maximum*^10^ and *Stylosanthes capitata* Vogel^11^. These studies evidenced that the injuries of water deficit in the stoichiometry result in nutritional imbalances, which could reflect in reduced dry matter production and nutrient accumulation in stems; however, these facts remain poorly investigated in sugarcane crops.

Thus, knowledge of stoichiometry is important because it involves the study of the balance of various chemical elements (especially C, N and P) in living systems^12^. Given the assimilation and interdependent use of several chemical elements essential to plants, it is imperative to maintain sufficient nutrient concentrations and stoichiometric balance in plant tissues for healthy growth, without inducing nutrient deficiency and toxicity^13^. In this context, the degree to which plants maintain a constant elemental composition in response to the availability of their environmental resources is called “stoichiometric homeostasis”^14^. Therefore, silicon (Si) may attenuate the stoichiometric disturbances of C:N:P in plant tissue that are caused by stress such as water deficit. This benefit of Si may be due to improved nutrient metabolism, as there are reports arguing that Si could replace part of the C in the epidermis cell wall as it binds to cellulose or hemicellulose compounds, favoring cell structuring, decreasing the demand for structural compounds such as lignin (which has a high energy cost for its synthesis)^15^, thus favoring the plant's energy savings, which may have an impact on the metabolism of other nutrients. In this context, the benefit of Si can be measured by evaluating the plant's ability to use C as well as N and P absorbed by the plant in its metabolism for conversion into dry mass. In other words, these nutritional benefits of Si could occur if the element contributed to increasing the efficiency of C, N and P use and, consequently, of plant growth. Therefore, according to Siddiqi and Glass^16^, the efficient use of a given nutrient is the capacity to produce dry mass per unit of cumulative nutrient in the plant.

Numerous strategies have been studied in the attempt of mitigating the damages caused by water deficit, and the use of Si stands out as one of the most relevant ones. Studies with Si in sugarcane are promising, especially when cultivated in sandy-textured soils, as they present a low available content of this beneficial element^17^, which requires the supply of Si to the plant. Another important point is the fact that crops accumulate this element in the aerial part^18,19^ because they have specific transporters for absorption of Si^20^. However, studies investigating Si use to mitigate negative effects caused by water deficit are restricted to sugarcane and directed to its physiological effects^6,21–23^. Thus, there is a knowledge gap regarding other approaches that investigate if Si could reduce severe water deficit by means of nutritional mechanisms, favoring the C:N:P stoichiometric homeostasis in stems of both sugarcane and energy cane.

To advance knowledge about the damage caused by water deficit in stoichiometric homeostasis and its relationship with Si, we hypothesized that (i) the damage caused by water deficit in the accumulation of dry matter by sugarcane and energy cane stems is due to a reduction in the absorption of N and P, associated to a modification in the C:N:P stoichiometry of stems, which in turn reduces the use efficiency of these macronutrients, and (ii) the provision of Si in both species could revert this damage caused by nutritional-water deficit and by the stoichiometric modification in stems, favoring the growth and development of plants.

The aims of this study were to evaluated the effects of water deficit in sugarcane and energy cane stems, in the absence and presence of Si, as well as its effects in the C:N:P stoichiometry of stems, in the use efficiency of N and P and in the accumulation of dry matter in stems.

## Results

### Si application increased the content of Si in stems of plants under water deficit and sufficiency

**Figure 1:**
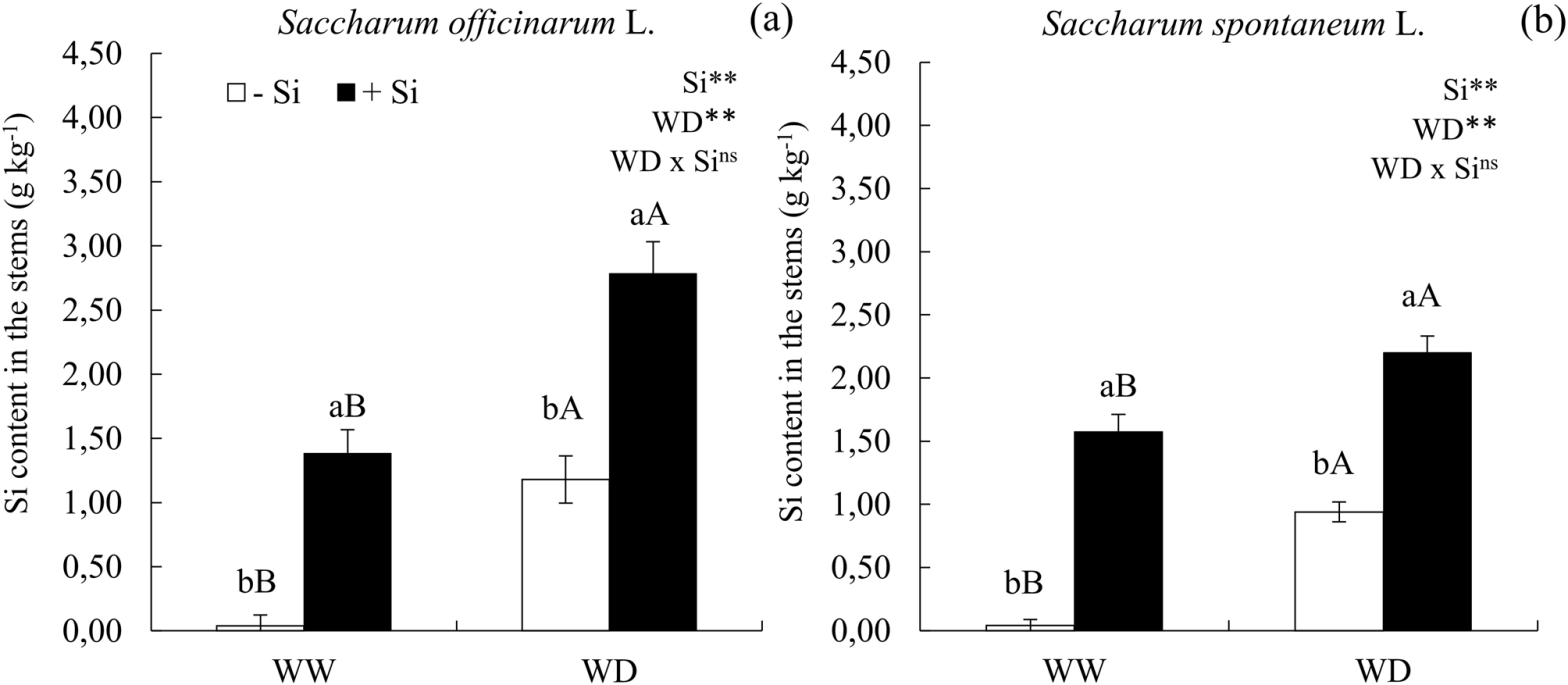
Silicon (Si) content in the stems of ratoon of sugarcane (*Saccharum officinarum* L.) (**a**) and energy cane (*Saccharum spontaneum* L.) (**b**) cultivated without water deficit-WW (70% of soil water retention capacity—WRC) and with water deficit-WD (30% of WRC) without (− Si) and with (+ Si) application of Si via fertigation associated with leaf spray. **Significant at 1% probability; and *ns* not significant for main effects and interaction, by the F-test. Lowercase letters show differences in relation to Si and uppercase letters in relation to WD. Bars represent the standard error of the mean. Si × WD: interaction.

The water deficit led to an increased concentration of Si in the stems of sugarcane (Fig 1a) and energy cane (Fig 1b). Si application via fertirrigation associated with foliar spraying increased the concentration of this element in the stems of sugarcane (Fig 1a) and energy cane (Fig 1b), in the presence and absence of a water deficit condition. The concentration of C, N and P are found in Supplementary Table S1.

### Water deficit reduced the accumulation of N and P in the stems, but the application of Si in a condition of water deficit increases N accumulation

**Figure 2:**
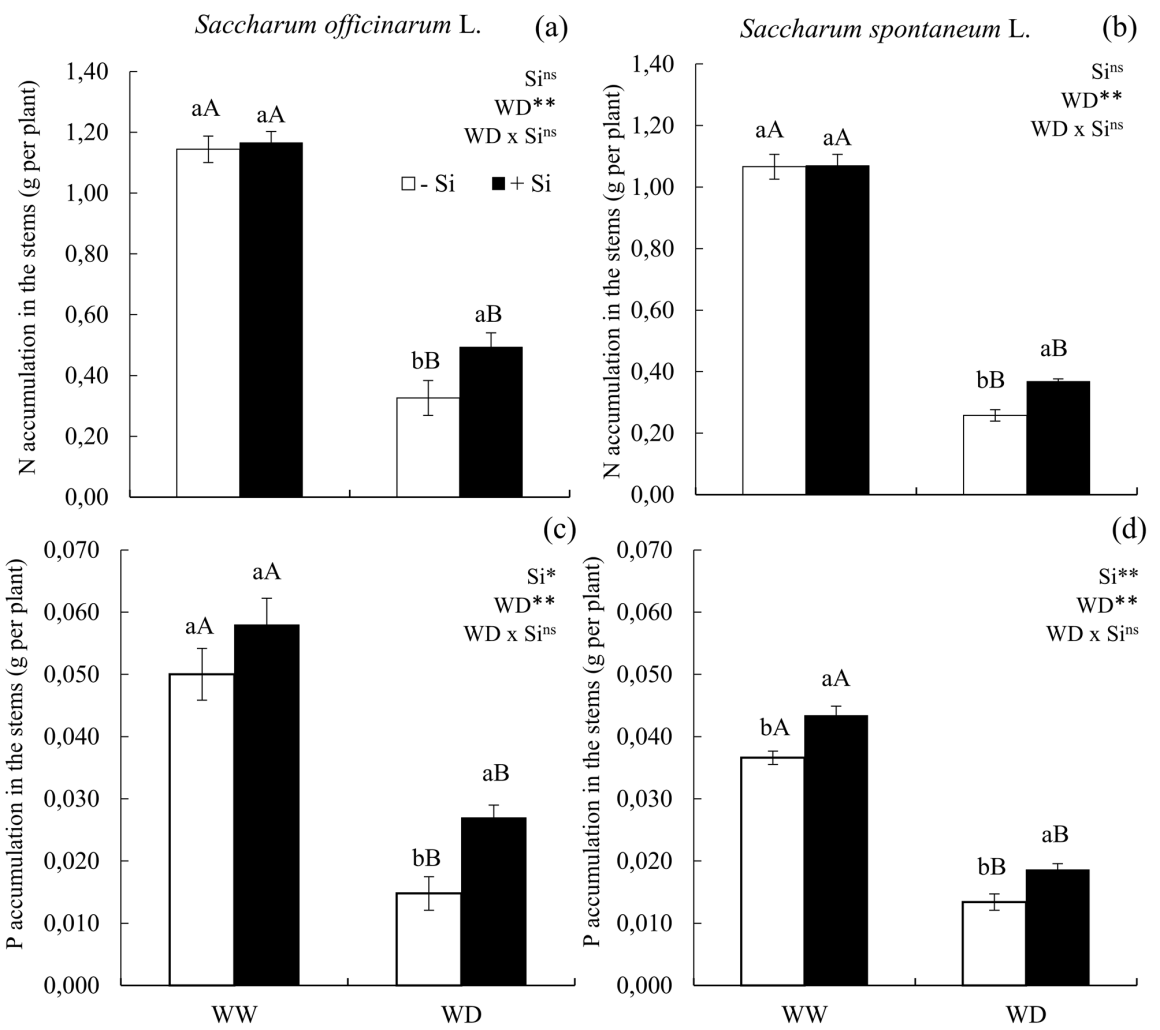
N accumulation (**a**,**b**) and P accumulation (**c**,**d**) in the stems of ratoon of sugarcane (*Saccharum officinarum* L.) (**a**,**c**) and energy cane (*Saccharum spontaneum* L.) (**b**,**d**) cultivated without water deficit-WW (70% of soil water retention capacity—WRC) and with water deficit-WD (30% of WRC) without (− Si) and with (+ Si) application of Si via fertigation associated with leaf spray. ** and *: significant at 1 and 5% probability, respectively; and *ns* not significant for main effects and interaction, by the F-test. Lowercase letters show differences in relation to Si and uppercase letters in relation to WD. Bars represent the standard error of the mean. Si × WD: interaction.

N accumulation in the stems was reduced in severe water deficit conditions in sugarcane (Fig 2a) and energy cane (Fig 2b). The application of Si in ratoons under water deficit increased N accumulation in the stems of sugarcane (Fig 2a) and energy cane (Fig 2b).

P accumulation in the stems was also reduced in water deficit conditions in sugarcane (Fig 2c) and energy cane (Fig 2d); however, the application of Si increased P accumulation in the stems of sugarcane under water deficit (Fig 2c), and in the stems of energy cane in both conditions (Fig 2d).

### Water deficit reduced the relations C:Si, C:N and C:P and the use efficiency of C, N and P in the stem

**Figure 3:**
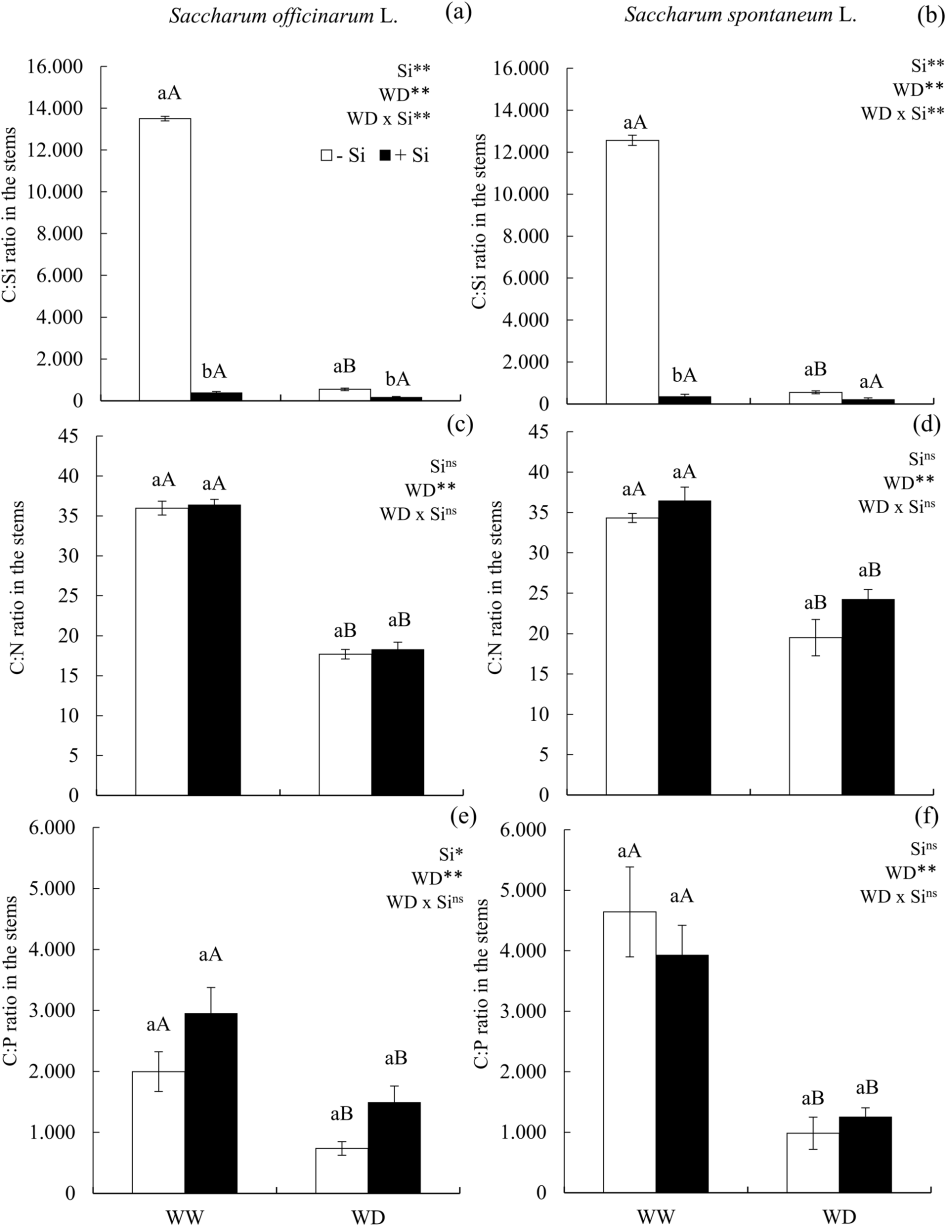
C:Si ration (**a**,**b**) C:N ration (**c**,**d**) and C:P ration (**e**,**f**) in the stems of ratoon of sugarcane (*Saccharum officinarum* L.) (**a**,**c**,**e**) and energy cane (*Saccharum spontaneum* L.) (**b**,**d**,**f**) cultivated without water deficit-WW (70% of soil water retention capacity—WRC) and with water deficit-WD (30% of WRC) without (− Si) and with (+ Si) application of Si via fertigation associated with leaf spray. ** and *: significant at 1 and 5% probability, respectively; and *ns* not significant for main effects and interaction, by the F-test. Lowercase letters show differences in relation to Si and uppercase letters in relation to WD. Bars represent the standard error of the mean. Si × WD: interaction.

The C:Si ratio of plants with and without Si application was reduced when these were found under water deficit, while in plants fertilized with this element, this ratio remained unchanged when the water deficit was imposed in sugarcane (Fig 3a) and energy cane (Fig 3b). The Si application with or without water deficit reduced the C:Si ratio in the stems of sugarcane (Fig 3a) and energy cane (Fig 3b).

Both C:N and C:P ratios of sugarcane (Fig 3c,e) and energy cane (Fig 3d,f) were reduced under water deficit conditions, in comparison to plants submitted to a water sufficiency condition (70% water retention capacity). The application of Si in both the presence and absence of water deficit did not increase the C:N and C:P ratios of sugarcane (Fig 3c,e) and energy cane (Fig 3d,f) stems.

**Figure 4:**
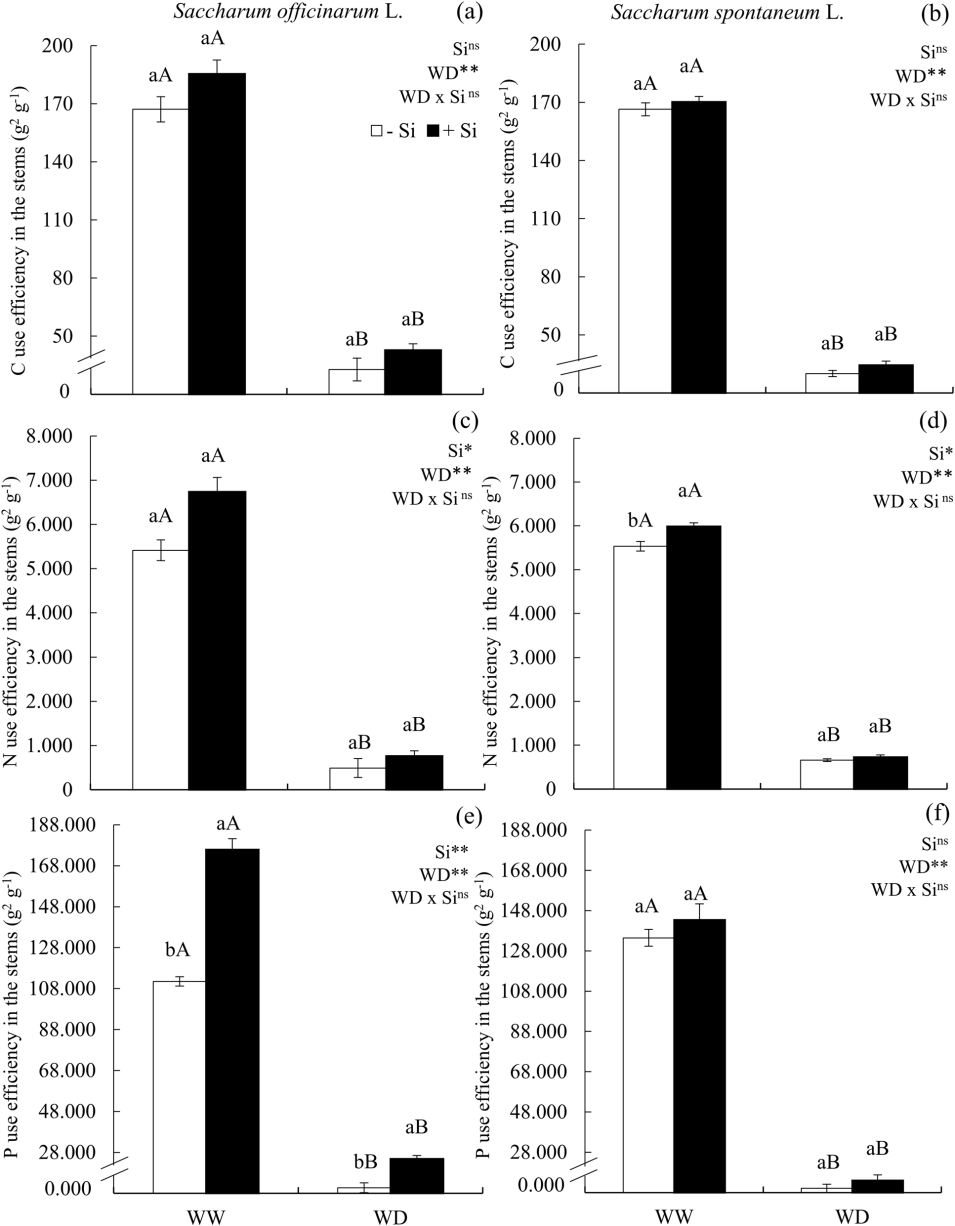
C use efficiency (**a**,**b**) N use efficiency (**c**,**d**) P use efficiency (**e**,**f**) in the stems of ratoon of sugarcane (*Saccharum officinarum* L.) (**a**,**c**,**e**) and energy cane (*Saccharum spontaneum* L.) (**b**,**d**,**f**) cultivated without water deficit-WW (70% of soil water retention capacity—WRC) and with water deficit-WD (30% of WRC) without (− Si) and with (+ Si) application of Si via fertigation associated with leaf spray. ** and *: significant at 1 and 5% probability, respectively; and *ns* not significant for main effects and interaction, by the F-test. Lowercase letters show differences in relation to Si and uppercase letters in relation to WD. Bars represent the standard error of the mean. Si × WD: interaction.

The use efficiency of C, N, and P was reduced in plants submitted to the condition of water deficit, in comparison to plants under water sufficiency, in sugarcane (Fig 4a,c,e) and energy cane (Fig 4b,d,f). Si application with and without a water deficit condition did not increase the use efficiency of C and N in sugarcane (Fig 4a,c,e), as well as the use efficiency of C and P of energy cane (Fig 4b,d,f). Both with and without water deficit, the application of Si resulted in an increased use efficiency of P in sugarcane (Fig 4e); however, without water deficit, Si led to an increased used efficiency of N in sugarcane (Fig 4d).

### Water deficit reduced the production of dry matter in stems, but Si attenuated the dry matter loss in the water deficit condition

**Figure 5:**
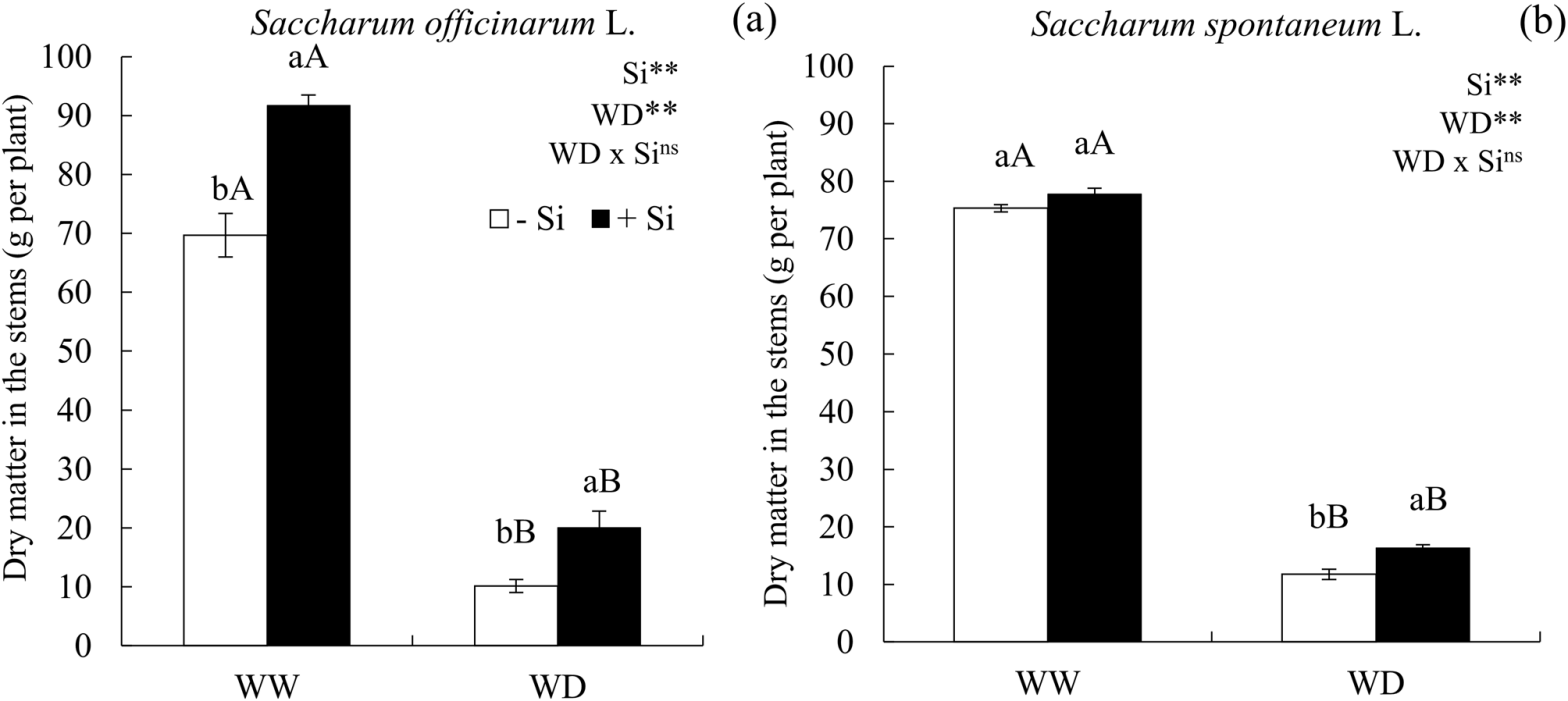
Dry matter of stems of ratoon of sugarcane (*Saccharum officinarum* L.) (**a**) and energy cane (*Saccharum spontaneum* L.) (**b**) cultivated without water deficit-WW (70% of soil water retention capacity—WRC) and with water deficit-WD (30% of WRC) without (− Si) and with (+ Si) application of Si via fertigation associated with leaf spray. ** and *: significant at 1 and 5% probability, respectively; and *ns* not significant for main effects and interaction, by the F-test. Lowercase letters show differences in relation to Si and uppercase letters in relation to WD. Bars represent the standard error of the mean. Si × WD: interaction.

The dry matter of sugarcane (Fig 5a) and energy cane (Fig 5b) stems decreased in plants submitted to a water deficit condition without Si. However, the plants under water deficit that received Si displayed an increased stem’s dry matter in sugarcane (Fig 5a) and energy cane (Fig 5b).

## Discussion

The benefit of Si in plants may depend on the absorption capacity of this element by the crop. Soils with low Si contents, such as the one used in this study (Typic Quartzipisamment) usually favor the effects of Si supply to plants, due to the low natural concentrations, which was evidenced because the Si content in the stems of plants submitted to the control treatment, with or without water deficit was low. The increased Si content in plants’ stems of both studied species, with or without a water deficit condition, occurred due to the quality of the sprayed solution, as its concentration (2.5 mmol L^−1^) was below the level known to trigger the polymerization of this element (3.0 mmol L^−1^), which would preclude its absorption^24^. Besides, the presence of the stabilizer sorbitol in the solution also reduced Si polymerization, by increasing the solution stability, as suggested by Kubicki and Heaney^25^, thus favoring the absorption of the element by plants. In addition, members of the Poaceae family are natural accumulators of Si by having specific transporters that enhance its absorption by crops^20^. It was demonstrated for the first time to the authors’ knowledge that when increasing Si contents in its stems in a similar way in comparison to sugarcane, energy cane plants might also have a good capacity of absorbing this element. However, absorption of Si in the stalk was not enough to indicate cane as an accumulator plant of the beneficial element, because the study organ, that is, the stalk of plants, presents lower content of Si.

Water deficit is one of the main causes affecting cane production^26^, seen that it hampers nutrients absorption. This effect of the water deficit was evidenced in this study, given the reduced accumulation of N and P in the stems of cane plants, due to its restricted mobility in the soil, which is needed to that the nutrients get in contact with roots. The contact between N and the roots occurs by the process of mass flow^27^, while P moves through diffusion^28^, but both macronutrients depend on the soil’s water availability. The reduced absorption of N and P in plants under water deficit was reported for other plant species, such as *Panicum maximum*^10^ and *Stylosanthes capitata* Vogel^11^.

The water deficit imposed in this study without Si application in cane plants promoted a stoichiometric modification in the stems of both species, by reducing the C:Si ratio. The lowest C:Si ratio occurred by the reduction in the C content in relation to the increase concentration of Si. Plants fertilized or not with Si in the condition of water deficit had their C:N and C:P ratios reduced, which might be justified by the effect of this water stress in the reduction of N and P contents, given the low absorption of these nutrients.

Modifications in the stoichiometric relations of C with Si, N and P caused by water deficit without Si supply, decreased the homeostasis of these elements in the plants’ stems, and consequently reduced its use efficiency. Plants under stress normally present strategies to promote a new stoichiometric homeostasis and balance their metabolism^29^. However, under severe conditions such as the one used in this study (maintaining the soil with 30% of the water retention capacity), these strategies were not efficient to preserve the balance of C, N and P in this organ of sugarcane and energy cane plants. Thus, it was evidenced for the first time that reduced accumulation of N and P, associated with a stoichiometric imbalance of C, N and P in stems and the low use efficiency of these nutrients promoted by the water deficit condition without Si, explains the low accumulation of biomass in these plants’ stems. It is noteworthy that the water deficit also affects the leaves as a result of the nutritional imbalance and physiological damage^6^, reducing the production of carbohydrates that would be remobilized to the stems, which in turn leads to a lower biomass accumulation in this organ.

In this study, it was evident that the biological damages caused by the condition of water deficit in sugarcane were similar to the ones observed in energy cane plants, but this is the first report for this species. These results allows us to accept the first hypothesis, indicating that the harm of water deficit in dry matter accumulation in sugarcane and energy cane stems was due to the reduced absorption of N and P, associated with a modification in the stoichiometry C:N:P in the stems, and reducing the use efficiency of these nutrients.

The importance of the water deficit in these species reinforces the need for strategies to mitigate such a prejudice, with Si being highlighted. In plants cultivated under water deficit, the supply of Si was important to promote N and P absorption. In the study performed by Gottardi et al.^30^, the authors indicated that the benefit of Si in increasing N absorption correlates with the regulation of genes transcription that are important codifiers for nitrate transporters. Its effect in P absorption were possibly due to the increase in the exudation of organic acids, which reduces the adsorption of inorganic P (Pi) in the rhizosphere, favoring the contact of the nutrient with the root and its radicular absorption^31^, in addition to the fact that Si occupies bonding sites of soil colloids and increases its availability in the soil^32^.

The benefic effect of Si in plants cultivated under water deficit regarding the modifications of stoichiometric relations was not clearly evidenced in the stems of sugarcane and energy cane plants. However, in conditions of water deficit, Si application was efficient in increasing the use efficiency of P in stems, but restricted to sugarcane. Although P is found in lower concentrations in the stems, this nutrient plays important roles in the plant’s metabolism, especially in protein formation and cell division^33^. In this sense, the increased use efficiency of this nutrient provided the highest photosynthetic rate, increasing the quantity of carbohydrates in the leaves, which was redistributed to stems^34^.

The benefit of Si in plants under water deficit was sufficient to increase dry matter production of stems of both species. These effects of Si in sugarcane can be explained by the highest absorption of N and P, and by the reduction of the C:Si ratio, which led to an increased use efficiency of P in stems. The reduction of the C:Si ratio might occur by decreasing the C content in stems, possibly because Si is immobilized in the cell wall in the form of phytoliths, a structural material that is similar to lignin^15^, which in turn reduces the demand for lignin, a fiber component that presents high energy cost for its biosynthesis, and might result in the reduction of the plant’s energy costs^35^^.^

Regarding energy cane plants, no effect of Si in the stoichiometry of C, N and P and in the use efficiency of these nutrients were observed, but a beneficial effect in the accumulation of dry matter in stems was found. This is an indicative that the mitigating effect of Si for plants under water deficit and in the C:N:P stoichiometry is influenced by genetic factors, highlighting the need for further research with this species. Therefore, the second hypothesis raised in this study is rejected, seen that it indicated that the Si supply in both studied species could revert these damages caused by water deficit, by means of stoichiometric modifications in stems, favoring the accumulation of biomass in this organ.

This study proposes for the first time that water deficit promotes a decreased production of sugarcane and energy cane stems, due to the stoichiometric imbalance of C, N and P, but the mitigating effect of Si in this organ is not related with this stoichiometric modification.

## Methods

Two experiments were performed in this study, being the first one with the ratoons of sugarcane variety RB966928; and the second with energy cane ratoons (variety VX2) cultivated in pots filled with a Typic Quartzipisamment, inside a greenhouse located at the São Paulo State University (UNESP), Campus of Jaboticabal. The stems were obtained from producers in the region of São Paulo. Our research was not conducted with endangered species and was conducted in accordance with the is in accordance with the Declaration of IUCN Policy on Research Involving Endangered Species.

During the production of pre-sprouted seedlings and throughout its growth, the plants received a Si application via fertirrigation, by means of five applications in a concentration of 2.5 mmol L^−1^.

A chemical analysis of the soil was performed before planting the cane plants in order to assess its fertility parameters, following the procedures proposed by Raij et al.^36^. The following results were obtained: pH (CaCl_2_) 4.3; organic matter 9 g dm^−3^; P resin 2 mg dm^−3^; Ca 3 mmol_c_ dm^−3^; Mg 1 mmol_c_ dm^−3^; K 0.3 mmol_c_ dm^−3^; Al 0 mmol_c_ dm^−3^; H + Al 16 mmol_c_ dm^−3^; sum of bases 4 mmol_c_ dm^−3^; cation exchange capacity (CEC) 20 mmol_c_ dm^−3^; bases saturation (K + Ca + Mg/K + Ca + Mg + H + Al) 21%. The Si content (1 mg dm^−3^) was determined by the method of Korndörfer et al.^37^.

During the implantation of cane plants, the soil was incubated with limestone (relative total neutralization power of 125%), in order to increase the bases saturation to 60%. A fertilization was performed with 150 mg dm^−3^ N (ammonium sulphate), P (triple superphosphate) and K (potassium chloride), 5 mg dm^−3^ Zn (zinc sulphate) and 2 mg dm^−3^ B (boric acid). After cutting the plants and in the beginning or regrowth of ratoons, a fertilization with 160 mg dm^−3^ of N (ammonium sulphate) and 60 mg dm^−3^ of K (potassium chloride) was performed.

Treatments were arranged in a factorial design 2 × 2 in both experiments, with two hydric conditions, being without water deficit (70% of the soil’s water retention capacity—WRC), and with water deficit (30% of the capacity), combined with either the presence (2.5 mmol L^−1^) or absence of Si applied via fertirrigation, associated with foliar spraying. Treatments were organized in a randomized blocks design with five replicates.

Water deficiency levels were determined according to the study of Teixeira et al. (2020), who reported that a water retention capacity of 70% could be considered an adequate condition, while a capacity of 30% can be considered a severe water deficit. The imposition of water deficit occurred 2 days after the first ratoons regrew, and was periodically controlled by weighting the pots and replacing the water lost by evapotranspiration^6^. The experimental plot was composed by polypropylene pots with a capacity of 20 dm^3^.

The source of Si used in these experiments was sodium and potassium silicate with sorbitol (113.4 g L^−1^ of Si, 18.9 g L^−1^ of K_2_O, 60.5 g L^−1^ of Na_2_O, and 20 mL L^−1^ of sorbitol, with a pH of 11.8). A concentration of 2.5 mmol L^−1^ was used in four applications, with the supply of Si was made by fertirrigation with soil application and by foliar spraying at 20, 35, 50 and 65 days after cutting the first ratoon, during the morning when the relative air moisture was 90, 86, 94 and 91%, and the temperature was 20.9, 21.5, 21.2 and 28.4, respectively.

Regarding the application of Si, a solution was prepared with its pH adjusted to 5.0 ± 0.2, with the aid of an HCl solution (1 mol L^−1^). The four application of Si via fertirrigation was performed by simulating a 5 mm blade of the silicate solution, while foliar spraying was made using a manual spray with a solution volume of 250 mL per plant. Foliar spraying was performed in order to guarantee that the leaf was entirely covered with the solution, without any runoff. The balance of the amount of K in the Si source was performed in plants that did not receive fertirrigation of this element, using a potassium chloride solution (1 mol L^−1^) at a concentration of 18.8 mg L^−1^ of K.

At 80 days after regrowth, the experiments were ended and the plants’ stems were collected. These samples were washed in tap water, then in a detergent solution (0.1% v/v), HCl solution (0.3% v/v) and in deionized water. The plant material was dried in an oven with forced air circulation (65 ± 5 °C) until constant weight, and then the dry matter was calculated. Subsequently, samples were milled in a Willey mill, and the Si content was determined by means of digesting the samples according to the methodology described by Kraska and Breitenbeck^38^. Colorimetric readings were performed according to the indications of Korndörfer et al.^37^.

The contents of C and N were determined through dry combustion (1000 °C), using the equipment LECO (Truspec CHNS), and the concentration of P was obtained from the nitric-perchloric digestion^39^. Based on the dry matter and in the concentrations of the elements, the accumulation of C, N and P was calculated in the stems. The relations C:Si, C:N, and C:P were calculated by the ration between the respective contents of these elements, and the use efficiency of each nutrient was calculated according to Siddiqi and Glass^16^, being ((total dry matter produced)^2^/(total accumulation of nutrients in the plant)). The obtained data were submitted to a variance analysis by the F test (p < 0.05) and means were compared by the Tukey’s test at a 5% probability level, using the software AgroEstat^40^.

## Supplementary Information

Supplementary Table S1.

## Data Availability

The datasets generated during and analysed during the current study are available from the corresponding author on reasonable request.
