# Supplementary material for: Water deficit modifies C:N:P stoichiometry affecting sugarcane and energy cane yield and its relationships with silicon supply
Source: Sci Rep. 2021 Oct 22;11:20916. doi: 10.1038/s41598-021-00441-0 (PMC8536714; doi:10.1038/s41598-021-00441-0)
Supplement: Supplementary file 1 — Supplementary Table S1. [file 41598_2021_441_MOESM1_ESM.docx]

# Water deficit modifies C:N:P stoichiometry affecting sugarcane and energy cane yield and its relationships with silicon supply

# Antonio Santana Batista de Oliveira Filho^1,*,a^, Renato de Mello Prado^1,b^, Gelza Carliane Marques Teixeira^1,c,+^, Marisa de Cássia Piccolo^2, d,+^, and Antonio Márcio Souza Rocha^3,e,+^

^1^ Department of Soils and Fertilizers, São Paulo State University (UNESP), Via de Acesso Prof. Paulo Donato Castellane, s/n, Jaboticabal, São Paulo 14884900, Brazil, antonio.filho@unesp.br^a^ ; rm.prado@unesp.br^b^ ; gelzacarliane@hotmail.com^c^, ^a^ORCID: 0000-0001-7398-0300, ^b^ORCID: 0000-0003-1998-6343, ^c^ORCID: 0000-0002-8062-482X

^2^ Center of Nuclear Energy in Agriculture (CENA), University of São Paulo (USP), Piracicaba, São Paulo, Brazil, mpiccolo@cena.usp.br^d^, ^d^ORCID: 0000-0003-2163-5630

^3^ Department of Technology, São Paulo State University (UNESP), Jaboticabal, São Paulo, Brazil, antonio.rocha@unesp.br^e^, ^e^ORCID: 0000-0003-0038-8503

^*^corresponding.author: antonio.filho@unesp.br. Telephone: +551632097404

^+^these authors contributed equally to this work

Supplementary Table S1: C, N and P concentration in the stems (g kg^-1^) of ratoon of sugarcane (*Saccharum officinarum* L.) (a) and energy cane (*Saccharum spontaneum* L.) (b) cultivated without water deficit-WW (70% of soil water retention capacity - WRC) and with water deficit-WD (30% of WRC) without (-Si) and with (+Si) application of Si via fertigation associated with leaf spray.

| *Saccharum officinarum* | | | | |
| --- | --- | --- | --- | --- |
|  | Without water deficit-WW | | With water deficit-WD | |
|  | +Si | -Si | +Si | -Si |
| C (g Kg^-1^) | 480,628 | 488,074 | 457,226 | 456,112 |
| N (g Kg^-1^) | 13,150 | 13,830 | 25,764 | 25,204 |
| P (g Kg^-1^) | 0,712 | 0,718 | 1,048 | 1,600 |
| *Saccharum spontaneum* | | | | |
|  | Without water deficit-WW | | With water deficit-WD | |
|  | +Si | -Si | +Si | -Si |
| C (g Kg^-1^) | 487,812 | 482,614 | 464,122 | 456,130 |
| N (g Kg^-1^) | 13,144 | 13,686 | 22,212 | 22,762 |
| P (g Kg^-1^) | 0,546 | 0,528 | 1,054 | 1,374 |
